# Prevalence Of, and Factors Associated with, Obesity among the Oldest Old. A Study Protocol for a Systematic Review

**DOI:** 10.3390/healthcare8030319

**Published:** 2020-09-04

**Authors:** André Hajek, Benedikt Kretzler, Hans-Helmut König

**Affiliations:** Department of Health Economics and Health Services Research, University Medical Center Hamburg-Eppendorf, 20246 Hamburg, Germany; b.kretzler.ext@uke.de (B.K.); h.koenig@uke.de (H.-H.K.)

**Keywords:** excess weight, overweight, obesity, adiposity, oldest old, aged, 80 and over, underweight, body-mass-index, systematic review

## Abstract

Some empirical studies have identified the prevalence of, and factors associated with, obesity among the oldest old. However, there is a lack of a systematic review synthesizing the existing evidence. Therefore, the purpose of our upcoming systematic review is to provide an overview of the evidence provided by observational studies. The current paper presents the protocol for this systematic review. We will search four electronic databases (Medline, PsycINFO, CINAHL, and Cochrane Library). Furthermore, we will perform a manual search (searching reference lists of included studies). Cross-sectional and longitudinal observational studies identifying the prevalence and preferably the factors associated with obesity among the oldest old (80 years and over) will be included. Data extraction will concentrate on study design, assessment of obesity and its associated factors, statistical analysis, sample characteristics, and key findings. We will evaluate the quality of the included studies. Two individuals will perform study selection, data extraction, and evaluation of study quality. We will present the results in figures, summary tables and narrative summaries. If data permits, a meta-analysis will be conducted.

## 1. Introduction

Excess weight (overweight and obesity) is an important risk factor for various chronic conditions [1]. Moreover, it is, among other things, associated with loneliness [2], poorer mental health [3], sickness absence (sick leave days and long-term absenteeism) [4], and increased health care use or costs [5]. In many countries prevalence rates of obesity are quite high [6]. 

Previous research has demonstrated that mean body weight continuously increases with age [7]. However, recent research has shown that the prevalence of obesity drops in individuals aged 75+ [8] (13.7% in total) or 85+ (oldest old: 10.2% in total) [9] in Germany. Similar prevalence rates for the oldest old were reported in Finland [10] and Canada [11], whereas higher prevalence rates were observed in Italy [12] and Spain [13]. Longitudinal studies, for example, have shown that the probability of obesity decreases with age [8,9], severe walking impairments [8] and less chronic conditions [9]. For example, the negative association between age and the probability of obesity can, among other things, be explained by the decrease in lean body mass (sarcopenia) [14]. Moreover, unintentional weight loss (as frailty component) is frequent in late life and can explain the link between age and obesity. 

While some empirical studies, as mentioned above, have identified the prevalence and correlates of obesity among the oldest old, there is a lack of a systematic review synthesizing the current evidence. Consequently, the goal of this systematic review is to give an overview of existing evidence based on observational studies. Knowledge about the prevalence of obesity among the oldest old is particularly important, given the number of people in this age bracket is expected to increase due to demographic aging. Furthermore, this review may identify risk factors for obesity. This knowledge may help to develop interventional strategies. Furthermore, a systematic review may assist in identifying potential gaps in research, which in turn may inspire future research in this area. 

This current study protocol is important because it documents the systematic review process before beginning the review and ensures the transparency of the final review [15]. More generally, it has been argued that study protocols for systematic reviews ensure that the following systematic reviews are planned in a careful way [15]. Additionally, such study protocols can be beneficial for accountability as well as the integrity of the research [15]. Moreover, problems can be anticipated, and readers can identify potential discrepancies between intended and actually-used methods. This can assist in determining potential biases in systematic reviews [15].

## 2. Materials and Methods

This study protocol for a systematic review was developed in line with the Preferred Reporting Items for Systematic Reviews and Meta-Analysis Protocols guidelines [15]. Additionally, it has been registered to the International Prospective Register of Systematic Reviews (PROSPERO, registration number: CRD42020193890).

### 2.1. Eligibility Criteria

A pretest of the inclusion criteria will be conducted (screening 100 titles/abstracts), and the inclusion criteria will be adapted if required. 

#### 2.1.1. Inclusion Criteria

Inclusion criteria for our systematic review are as follows:

- cross-sectional and longitudinal observational studies examining the prevalence of obesity among the oldest old (at least 80 years and over), and ideally (but not necessarily) determining its associated factors
- assessment of key variables with appropriate tools
- studies in English or German language, published in peer-reviewed, scientific journal

It should be noted that the oldest old are often defined either as being 80 years and over (e.g., the respective MeSH-Term (MeSH: Medical Subject Headings) or empirical studies such as [16,17]) or 85 years and over (e.g., [18,19]). By including individuals 80 years and older, we will follow a rather broad and inclusive search strategy in our upcoming systematic review. 

#### 2.1.2. Exclusion Criteria 

Exclusion criteria for our systematic review are:

- studies solely investigating samples with a specific disorder (e.g., individuals with mental disorders).

Electronic databases (Medline, PsycINFO, CINAHL, and Cochrane Library) will be searched using predefined search terms. The Medline search strategy is displayed in Table 1. In addition, the reference lists of included studies will be searched manually. No restrictions will be applied to the search with regard to the time and location of publication.

### 2.2. Data Management 

Endnote X7 (Clarivate Analytics, Philadelphia, PA, USA) will be used for data import. Moreover, Stata 16.0 (StataCorp, College Station, TX, USA) will be used for meta-analysis (using the ‘meta summarize’ command), if possible. 

### 2.3. Study Selection Process 

A title/abstract screening (first step) will be conducted by two reviewers (A.H., B.K.). In the second step, these two reviewers will review the full texts. Discussion will be used to resolve any difference in opinion between the two reviewers. If agreement cannot be reached, a third party (H.-H.K.) will be consulted. 

### 2.4. Data Collection Process and Data Items 

Data extraction will be undertaken by two reviewers (A.H., B.K.). One reviewer will extract the data and a second reviewer will perform a cross-check (data: study design, definition and measurement of main variables (i.e., obesity), sample characteristics, statistical analysis, and key findings). If discrepancies occur, a third party (H.-H.K.) will be involved. Study authors will be contacted when relevant data cannot be extracted, or where clarification is required.

### 2.5. Assessment of Study Quality/Risk of Bias 

We will use an appropriate tool to evaluate the quality of the included studies, such as the NIH Quality Assessment Tool for Observational Cohort and Cross-Sectional Studies [20]. Two reviewers (A.H. and B.K.) will perform the quality assessment. If required, discussions will be held to reach a consensus. A third party (H.-H.K.) will be involved, if necessary. 

### 2.6. Data Synthesis 

A PRISMA (Preferred Reporting Items for Systematic Reviews and Meta-Analyses) flow chart will be used to display the process of study selection. Moreover, a narrative synthesis will outline the studies’ main findings. 

If possible, results will be categorized according to continent (i.e., Europe, Asia, Africa, North America, South America and so on) or region, or according to the measurement of obesity.

If data permit, a meta-analysis will be performed. More precisely, in such a case, random effects meta-analysis will be conducted. The underlying assumption is that the study effect sizes differ and that the studies collected reflect a random sample from a larger population of studies [21]. 

### 2.7. Patient and Public Involvement Statement 

The present review protocol did not involve individual patients or public agencies.

## 3. Discussion

While some previous studies [8,9,10,11,12,13] have reported the prevalence of obesity among the oldest old, no study has systematically synthesized the prevalence of obesity, and the factors associated with it, among the oldest old. Therefore, our aim is to close this gap in knowledge. A further aim is to assess study quality. Additionally, our systematic review may reveal important (modifiable) risk factors for obesity in very late life, which in turn may be important for informing interventions. 

More precisely, previous studies reported prevalence rates of obesity of about 10% among the oldest old in Germany [9], Finland [10] and Canada [11]. In contrast, somewhat higher prevalence rates were documented in Spain (women: 29.2%, men: 19.4%) and Italy (women: 25.4%, men: 7.7%). Factors associated with a higher likelihood of obesity among the oldest old include, among other things, more chronic conditions [9] or limitations in daily living activities [10]. Chronic conditions such as arthrosis can reduce the mobility of individuals, which in turn can contribute to obesity [22]. However, other chronic conditions such as cancer are linked to a lower probability of obesity due to the weight loss associated with this disease. Thus, the link between chronic conditions and obesity is complex and requires further attention by using, for example, multimorbidity clusters [9]. Beyond these findings, it is worth noting that a sedentary lifestyle is a key factor contributing to obesity in somewhat younger age groups [22]. 

The link between obesity and other geriatric syndromes such as visual and hearing impairment, falls or dementia has rarely been investigated exclusively among the oldest old (e.g., [9]), and is therefore poorly understood in this age bracket. Previous research focusing on old age (instead of the oldest old) showed that higher body weight can be protective for geriatric syndromes such as frailty [23]. However, it should be noted that the link between these factors is complex and mixed evidence exists [24]. Therefore, future research is required to disentangle the link between obesity and geriatric syndromes exclusively focusing on the oldest old. 

### Strengths and Limitations

To the best of our knowledge, this will be the first systematic review focusing on the prevalence and factors associated with obesity among the oldest old. Important procedures of the review, such as the study selection, data extraction and the evaluation of study quality will be performed by two reviewers. If requirements are met, a meta-analysis will be conducted. 

## 4. Conclusions

Our systematic review may identify possible gaps in knowledge, such as a lack of studies including both individuals residing in the community, as well as those residing in institutionalized settings. Additionally, and more generally, there may be a lack of samples collected from the general population in old age. Moreover, existing studies may mainly rely on self-assessments of both weight and height, which could emphasize the need for future studies based on objective assessments (e.g., measured waist circumference). Furthermore, existing studies may focus on European countries (for example: [8,9,10,11,12,13]), whereas studies from other regions (e.g., Asian or Africa) may be missing. In addition, previous studies may be mainly based on cross-sectional data, with few studies exploiting longitudinal data. In sum, determining these potential gaps in research may encourage more research in this important field. 

## 5. Ethics and Dissemination

No primary data will be collected. Therefore, approval by an ethics committee is not required. We plan to publish our findings in a peer-reviewed journal. 

## Figures and Tables

**Table 1:** Search strategy (Medline).

Medline Search Algorithm:	
#1	Overweight
#2	Excess weight
#3	Obes *
#4	Adipos *
#5	#1 OR #2 OR #3 OR #4
#6	Oldest old
#7	Octogenarian
#8	Aged, 80 and over [MeSH Terms]
#9	#6 OR #7 OR #8
#10	Prevalence [Title/Abstract]
#11	#5 AND #9 AND #10

Note: The asterisk (*) is a truncation symbol.
